# Adverse Events After Metastases-Directed Stereotactic Radiotherapy and Biological Cancer Therapy

**DOI:** 10.1001/jamanetworkopen.2025.53809

**Published:** 2026-01-14

**Authors:** Esmée L. Looman, Stephanie G. C. Thoma, Jana Schaule, Mathieu Spaas, Klaus Henning Kahl, Joost J. C. Verhoeff, Famke L. Schneiders, Oliver Blanck, Fabian Lohaus, Susanne Rogers, David Kaul, Sergi Benavente, Stephanie E. Combs, Georgios Skazikis, Kynann P. Aninditha, Ilinca Popp, Friederike L. A. Koppe, Hans Geinitz, Katrien de Jaeger, Shankar Siva, Susanne Stera, Andrea Wittig, Victor Lewitzki, Franziska Eckert, Markus M. Schymalla, Matthias Guckenberger

**Affiliations:** 1 University Hospital Zürich, Radiation Oncology, Zürich, Switzerland; 2 University of Zürich, Zürich, Switzerland; 3 Cantonal Hospital Aarau, Radiation Oncology Center Mittelland, Aarau, Switzerland; 4 Ghent University Hospital, Radiation Oncology, Ghent, Belgium; 5 Universitätsklinikum Augsburg, Radiation Oncology, Augsburg, Germany; 6 University Medical Center Utrecht, Radiation Oncology, Utrecht, the Netherlands; 7 Amsterdam UMC location Vrije Universiteit Amsterdam, Radiation Oncology, Amsterdam, the Netherlands; 8 University Medical Center Schleswig-Holstein, Radiation Oncology, Kiel, Germany; 9 Technische Universität Dresden, Radiation Oncology, Dresden, Germany; 10 National Center for Tumor Diseases, Faculty of Medicine, University Hospital Carl Gustav Carus, Dresden University of Technology, Dresden, Germany; 11 OncoRay-National Center for Radiation Research in Oncology, Faculty of Medicine, University Hospital Carl Gustav Carus, Dresden University of Technology and Helmholtz-Zentrum Dresden-Rossendorf, Dresden, Germany; 12 German Cancer Consortium (DKTK), Partner Site Dresden, Dresden, Germany; 13 Department of Radiation Oncology, University Hospital Carl Gustav Carus, Dresden, Germany; 14 Charité-University Hospital Berlin, Radiation Oncology, Berlin, Germany; 15 Health and Medical University Potsdam, Radiation Oncology, Potsdam, Germany; 16 Vall d’Hebron University Hospital Campus, Radiation Oncology, Barcelona, Spain; 17 Technical University Munich, Radiation Oncology, Munich, Germany; 18 Schwarzwald-Baar Klinikum, Radiation Oncology, Villingen-Schwenningen, Germany; 19 Klinikum Stuttgart, Radiation Oncology, Stuttgart, Germany; 20 Department of Radiation Oncology, Medical Center–University of Freiburg, Faculty of Medicine, Freiburg, Germany; 21 Instituut Verbeeten, Radiation Oncology, Tilburg, the Netherlands; 22 Ordensklinikum Linz, Radiation Oncology, Linz, Austria; 23 Johannes Kepler Universität Linz, Medizinische Fakultät, Linz, Austria; 24 Catharina Hospital, Radiation Oncology, Eindhoven, the Netherlands; 25 Peter MacCallum Cancer Centre and the University of Melbourne, Radiation Oncology, Melbourne, Australia; 26 University Hospital Frankfurt, Radiation Oncology, Frankfurt, Germany; 27 National Center for Tumor Diseases, University Hospital Würzburg, Würzburg, Germany; 28 Department of Radiotherapy and Radiation Oncology, University Hospital Würzburg, Würzburg, Germany; 29 University Hospital Jena, Radiotherapy and Radiation Oncology, Jena, Germany; 30 University Hospital Tübingen, Radiation Oncology, Tübingen, Germany; 31 Medical University Vienna, Radiation Oncology, Vienna, Austria; 32 Philipps-University Marburg, Radiation Oncology, Marburg, Germany

## Abstract

**Importance:**

Metastases-directed stereotactic radiotherapy (SRT) is increasingly performed in patients with metastatic or oligometastatic cancer treated with immune checkpoint inhibitors (ICIs), monoclonal antibodies (mAbs), and small-molecule drugs (SMs). However, little is known about potential interactions between SRT and biological cancer therapy (BCT).

**Objective:**

To prospectively investigate adverse events associated with SRT combined with concurrent BCT.

**Design, Setting, and Participants:**

This international, prospective, multicenter, noninterventional registry cohort study (Toxicity and Efficacy of Combined Stereotactic Radiotherapy and Systemic Targeted or Immune Therapy [TOaSTT]) was conducted between July 2017 and August 2019 with a 24-month follow-up. Patients from 27 centers whose cancer was treated with metastases-directed SRT concurrently with BCT were eligible. Analyses were performed in January 2025.

**Exposure:**

Patients treated with SRT for intracranial or extracranial metastases and concurrent (within ≤30 days) BCT. Indication for treatment, decision on the radiotherapy dose and fractionation, as well as interruption of BCT, were left to the discretion of the treating clinician.

**Main Outcomes and Measures:**

The primary outcome was severe (at least grade 3) adverse events of combined modality treatment, as graded by the treating physician. Overall survival (OS) and progression-free survival (PFS) were secondary end points.

**Results:**

In total, 514 SRTs (271 cranial and 243 extracranial) concurrent with BCT were performed in 433 patients (median [IQR] age, 62 [54-70 years; 275 male [63.5%]). In 315 SRTs (61.3%) patients received ICIs, whereas in 150 SRTs (29.2%), patients received SMs and in 49 SRTs (9.5%) patients received mAbs. In 430 SRTs (83.7%), BCT had been initiated in patients before SRT, while 71 of 392 patients (18.1%) paused BCT during SRT. Severe (≥grade 3) acute adverse events were observed in 27 of 506 treatments (5.3%; 3 patients with grade 5 events), and severe late adverse events were observed in 29 of 459 patients (6.3%; 2 patients with grade 5 events). SRT with uninterrupted BCT was not associated with increased severe acute or late adverse events (odds ratio, 2.32; 95% CI, 0.87-6.22). Interruption of BCT during SRT was not associated with worse PFS and OS after correction for performance status and histologic type (hazard ratio, 0.81; 95% CI, 0.61-1.09; *P* = .17).

**Conclusions and Relevance:**

In this cohort study of 433 patients, severe adverse events after SRT and concurrent BCT were uncommon (<10%), continuing BCT during SRT was not associated with increased risk of severe adverse events, and interrupting BCT was not associated with worse OS when correcting for patients’ performance status. These findings suggest a favorable safety profile of metastases-directed SBRT in combined modality treatment settings.

## Key Points

**Question:**

What adverse events are associated with metastases-directed stereotactic radiotherapy (SRT) with concurrent biological cancer therapy (BCT) in patients with metastatic or oligometastatic cancer?

**Findings:**

This cohort study, including 433 patients and 514 SRTs, observed low risk (<10%) of severe acute or late adverse events following concurrent treatment with SRT and BCT. Uninterrupted treatment with BCT during SRT was not associated with significantly increased severe adverse events.

**Meaning:**

These results suggest that the favorable safety profile of metastases-directed SRT persists in combined modality treatments and provide exploratory insights into the safety profiles of SRT across different anatomical sites with concurrent BCT.

## Teaser

This cohort study examines adverse events associated with stereotactic radiotherapy combined with concurrent biological cancer therapy in patients with metastatic or oligometastatic cancer.

## Introduction

Patients with metastatic cancer are increasingly treated with biological cancer therapy (BCT), including immunotherapy and targeted therapies.^1^ Immune checkpoint inhibitors (ICIs), monoclonal antibodies (mAbs), and small-molecule (SM) anticancer drugs have become standard treatment options by achieving prolonged survival and/or disease control in many cancers.^2,3^ Resistance development to BCT frequently leads to oligoprogressive disease, and progression is often observed in initially involved sites^4,5,6,7,8^; this forms the rationale for a multidisciplinary treatment approach of metastases-directed stereotactic radiotherapy (SRT) added to systemic BCT, aimed at overcoming localized drug resistance.^9,10^

Metastases-directed SRT has been shown to achieve durable local control of treated metastases, leading to improved progression-free survival (PFS) and delayed switch of systemic therapy.^9,11,12,13,14,15^ However, limited data are available regarding the safety profile of combined BCT with SRT, and this knowledge gap is even more evident with respect to prospective evidence and individualized drug-SRT combinations.^16,17^ While there is emerging prospective evidence on the safety profile of combined SRT with ICIs, recent systematic reviews revealed a lack of data for many BCTs, as well as various anatomical sites of SRT.^16,18,19,20,21^ This challenge will further increase due to rapid and continuous approval of new BCTs and increasing heterogeneity of tumor types regarding molecular and genetic subclassification.^22^ This makes it challenging, if not impossible, to obtain sufficient prospective randomized data for all possible treatment combinations in clinical practice. To address this question, we conducted a prospective multicenter registry study to generate exploratory data about the safety profile of combining metastases-directed SRT and BCT in patients with metastatic cancer.

## Methods

### Design, Setting, and Participants

This cohort study used data from Toxicity and Efficacy of Combined Stereotactic Radiotherapy and Systemic Targeted or Immune Therapy, an international multicenter prospective registry study aiming to investigate the safety profile of metastases-directed SRT for patients concurrently receiving BCT (ICIs, mAbs, or SMs). All patients with cancer who were treated with metastases-directed SRT concurrently with BCT were eligible for enrollment, with concurrent defined as systemic therapy given within 30 days before or after SRT. A single definition of concurrent treatment of 30 days was pragmatically chosen based on the half-life of many commonly used BCTs (eg, aPD1 [ anti-programmed death-1]: 20-30 days).^23^ Interruption of BCT was reported by the treating clinicians when 1 or more BCT application was withheld due to toxic effect concerns. Cranial SRT was defined as the delivery of fractionated stereotactic radiotherapy in 5 or fewer fractions or single-fraction radiosurgery. Extracranial SRT was defined as delivery of maximum 10 fractions with a minimum total biologically effective dose with an α/β ratio of 10 Gy (BED 10) of 50 Gy; a minimum total dose of 45 Gy was accepted as a minor deviation.^24,25^ Imaging for target delineation was performed according to the clinics’ standard of care.^26^ Patients treated in a sequential approach, with only cytotoxic chemotherapy or antihormonal therapy, and patients treated with non-SRT were excluded. The expected enrollment was 375 patients without a formal sample size calculation. The study was open for patient enrollment for 24 months between July 2017 to August 2019, and data acquisition continued for 24 months after enrollment of the last patient (total follow-up, 24 months). Follow-up visits took place every 3 months in the form of clinical visits. The baseline for follow-up was the date of SRT initiation because SRT-related adverse events, including potential Common Terminology Criteria for Adverse Events Grade 5 adverse events affecting overall survival (OS) could occur from the moment of SRT initiation. This was a noninterventional observational registry study designed to collect detailed exploratory data on the safety profile of concurrent metastases-directed SRT and BCT, in which treatment indication, radiotherapy dose, and fractionation, as well as the decision to interrupt BCT and the duration of such interruption, were left to the discretion of the treating clinician. Ethics committee approval was obtained from all participating sites. Written informed consent was signed by all study participants. Reporting follows the Strengthening the Reporting of Observational Studies in Epidemiology (STROBE) reporting guideline.

### Outcomes

The primary end point of this study was severe (at least grade 3) adverse events of combined modality treatment, as graded by the treating physician, in the overall patient population. Exploratory analyses were conducted with respect to individual drugs and anatomical location of SRT-treated metastases. OS was a secondary end point. Acute adverse events (developing within 3 months following SRT) and late adverse events (still existing or developing 3 months or later after SRT; follow-up of 24 months) were evaluated using Common Terminology Criteria for Adverse Events version 4.03.^27^ Adverse events were recorded both per patient and per treatment course, in case patients were treated for multiple localizations. Adverse events were graded as a binary end point.

Adverse events were recorded if it was attributed to the combination of SRT and BCT; this was any adverse event within organs or anatomical compartments that were exposed to radiation. Radiation dose was calculated as BED in Gy. An α/β ratio of 10 Gy was utilized for acute adverse events and an α/β ratio of 3 Gy for late adverse events. OS was defined as the time from SRT to death or last follow-up. Follow-up was performed according to the participating clinics’ clinical practice.

### Statistical Analysis

Statistical analyses were conducted using R version 4.3.2 in January 2025. Baseline differences in Eastern Cooperative Oncology Group Performance Status (ECOG-PS) and Charlson comorbidity scores between patients with and without concurrent systemic therapy were analyzed using the Wilcoxon rank-sum test. Rates of severe acute and late adverse events are reported. Additionally, we calculated the prevalence of severe adverse events for different follow-up time points. We performed an exploratory descriptive analysis of the occurrence of severe acute and late adverse events for each BCT separately and for the SRT locations (brain, thorax, abdomen, pelvis, and other). Logistic regression was used for hypotheses-generating analyses of possible exposures for severe adverse events. Kaplan-Meier survival curves with log-rank analysis and Cox proportional hazards regression model were utilized to assess OS for patients continuing BCT during SRT vs no BCT during SRT. A 2-sided *P* < .05 was considered statistically significant.

## Results

### Patient Characteristics

From July 2017 to August 2019, 514 metastases-directed SRT procedures were performed in 433 patients with metastatic cancer across 27 centers (Table 1 and Table 2). Patients had a median (IQR) age of 62 (54-70) years, were mostly male (275 patients [63.5%]), and most frequently had a diagnosis of malignant melanoma (160 patients [37.0%]) or non–small cell lung cancer (155 patients [35.8%]). Most patients (431 patients [99.5%]) had undergone prior local or systemic cancer therapy. Most patients had a good clinical performance status (ECOG-PS 0-1, 388 patients [89.6%]), but a majority had multiple comorbidities (age-adjusted Charlson comorbidity score ≥3, 250 patients [57.7%]). Patients treated with concurrent BCT had better ECOG PS compared with those who did not receive concurrent BCT (193 of 312 patients [61.9%] vs 66 of 140 patients [47.1%]; ECOG 0: 0 patients vs ECOG 3 or 4: 2 patients [1.4%]; *P* = .004). Oligometastatic disease (≤5 lesions) was present in 182 patients (42.0%). Median (IQR) follow-up for all patients was 18 (8-25) months.

**Table 1.** Patient Characteristics

Patient characteristics	Patients, No. (%) (N = 433)
Age, median (IQR), y	62 (54-70)
Sex	
Female	158 (36.5)
Male	275 (63.5)
Primary tumor	
Melanoma	160 (37.0)
Non–small cell lung cancer	155 (35.8)
Renal cell carcinoma	37 (8.5)
Breast cancer	25 (5.8)
Bladder cancer	25 (5.8)
Other	31 (7.2)
ECOG-PS prior to SRT treatment	
0-1	388 (89.6)
2-4	31 (7.2)
Unknown	14 (3.2)
Age-adjusted Charlson Comorbidity Index Score	
<3	183 (42.3)
≥3	250 (57.7)
Steroids during SRT	
Yes	105 (24.2)
No	318 (73.4)
Unknown	10 (2.3)
Metastatic state	
Oligometastatic (≤5 lesions)	182 (42.0)
Polymetastatic (>5 lesions)	222 (51.3)
Unknown	29 (6.7)
Involved organs	
1	126 (29.1)
>1 (2-6)	287 (66.3)
Unknown	20 (4.6)

Abbreviations: ECOG-PS, Eastern Cooperative Oncology Group Performance Status; SRT, stereotactic radiotherapy.

**Table 2.** Treatment Characteristics

Treatment characteristics	Treatments, No. (%) (N = 514)
Cranial SRT	
Treatments, No.	271
Gross tumor volume, median (IQR), mL	2.00 (0.56-6.80)
Radiation dose BED10, median (IQR), Gy	62 (60-88)
Radiation dose BED3, median (IQR), Gy	153 (118-233)
Fractions, No./total No. (%)	
1	195/283 (68.9)
2-5	88/283 (31.1)
Extracranial SRT	
Treatments, No.	243
Gross tumor volume, median (IQR), mL	10 (5-31)
Radiation dose BED10, median (IQR), Gy	70 (54-107)
Radiation dose BED3, median (IQR), Gy	137 (100-218)
Fractions, No./total No. (%)	
≤5	190/236 (80.5)
6-10 fractions	46/236 (19.5)
Systemic	
Concurrent chemotherapy	41 (8.0)
Biological cancer therapy during SRT	
Yes	354 (68.8)
No (treatment interrupted or started after SRT)	156 (30.4)
Unknown	4 (0.8)
Immune checkpoint inhibitors	
aPD-(L)1	270 (52.5)
aPD-(L)1 and aCTLA-4	45 (8.8)
Monoclonal antibodies or small molecules	
BRAF/MEKi	40 (7.8)
aEGFR/EGFRi	49 (9.5)
mTKI	33 (6.4)
ALKi	18 (3.5)
aVEGF	17 (3.3)
aHER2/HER2i	22 (4.3)
Other	20 (3.9)

Abbreviations: aCTLA-4, anticytotoxic T-lymphocyte-associated protein 4; aEGFR, anti–epidermal growth factor receptor; aHER2, anti–human epidermal growth factor receptor 2; ALKi, anaplastic lymphoma kinase inhibitor; aPD-(L)1, anti-programmed death-ligand 1 antibody; aVEGF, antivascular endothelial growth factor; BED, biological equivalent dose; BRAF/MEKi, BRAF/MEK-inhibitor; EGFRi, epidermal growth factor receptor inhibitor; HER2i, human epidermal growth factor receptor 2 inhibitor; mTKI, multitargeted tyrosine kinase inhibitor; SRT, stereotactic radiotherapy.

### Treatment Characteristics

In 315 of 514 cases (61.3%), patients received ICIs, 150 (29.2%) received SMs, and 49 (9.5%) received mAbs (Table 2). In most cases (430 patients [83.7%]), patients had initiated BCT before SRT, with a median (IQR) lead time of 105 (20-306) days before SRT. Of 392 patients, 71 (18.1%) interrupted their BCT during SRT; of these interrupted treatments, 41 (57.7%) consisted of tyrosine kinase inhibitors. If interrupted, BCT was paused for a median (IQR) of 13 (6-19) days before SRT in patients receiving ICIs, 18 (9-35) days when receiving mAbs, and 3 (1-5) days when receiving SMs. After SRT, BCT was restarted after a median (IQR) of 9 (7-13) days for ICIs, 8 (7-16) days for mAbs, and 3 (1-4) days for SMs. BCT was started after SRT in 85 patients (16.5%), and the median time interval was 10 days (IQR 5-15 days) following SRT.

Most SRT treatments (271 treatments) were targeting intracranial metastases. The median (IQR) number of intracranial metastases per treatment course was 2 (1-3), with a cumulative median (IQR) gross tumor volume of 2.0 (0.6-6.8) cm^3^. The prescribed median (IQR) SRT dose (BED10) to the intracranial planning targeted volume was 62 (60-88) Gy in a median of 1 fraction. Of all SRTs, 243 targeted extracranial metastases. The median (IQR) number of extracranial metastases per treatment course was 1 (1-2) metastasis. The cumulative median gross tumor volume for extracranial metastases was 10 (5-31) cm^3^, with a median (IQR) extracranial prescribed SRT dose (BED10) of 70 (54-107) Gy (Table 2).

### Grade 5 Adverse Events (Patient Death)

Cumulatively, grade 5 adverse events (patient death) occurred in 5 of 433 patients (1.2%). Acute grade 5 adverse events were observed in 3 of 506 patients (0.6%), and late grade 5 adverse events were observed in 2 of 459 patients (0.4%) (eTable 1 and eTable 2 in Supplement 1). All grade 5 adverse events were observed after SRT for cranial metastases in patients who continued BCT during SRT (ICIs, 2 patients; BRAF/MEK inhibitor [BRAF/MEKi], 3 patients). Grade 5 adverse events consisted of intracranial hemorrhage (3 patients BRAF/MEKi and anti-programmed death-ligand 1 [aPD-(L)1]), central nervous system necrosis combined with intracranial hemorrhage (1 patient; BRAF/MEKi) and other or unknown (1 patient; aPD-(L)1) (eTable 1 and eTable 2 in Supplement 1).

### Acute Adverse Events

**Figure 1.**
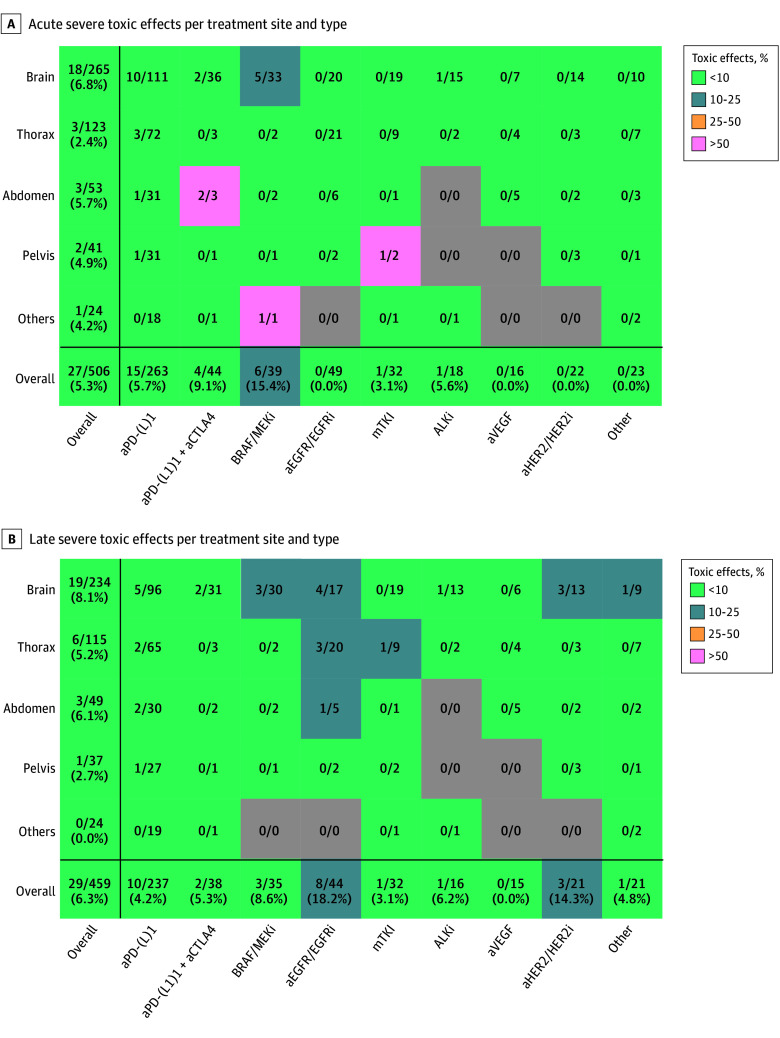
Observed Acute and Late Severe Toxic Effects After Stereotactic Radiotherapy Per Treatment Site and Biological Cancer Therapy Type The figure shows the number of patients that experienced severe acute (≤3 months; A) or late (>3 months; B) adverse events for combinations of stereotactic radiotherapy treatment sites and biological cancer therapy types and the total number of patients that were exposed to this multimodality treatment combination. The percentage of severe adverse events per combination is calculated and color-coded. aCTLA-4 indicates anticytotoxic T-lymphocyte-associated protein 4; aEGFR, anti–epidermal growth factor receptor; aHER2, anti–human epidermal growth factor receptor 2; ALKi, anaplastic lymphoma kinase inhibitor; aPD-(L)1, anti-programmed death-ligand 1 antibody; aVEGF, antivascular endothelial growth factor; BRAF/MEKi, BRAF/MEK-inhibitor; EGFRi, epidermal growth factor receptor inhibitor; HER2i, human epidermal growth factor receptor 2 inhibitor; mTKI, multitargeted tyrosine kinase inhibitor.

Severe acute (≥ grade 3) adverse events were observed in 27 of 506 concomitant SRT treatments (5.3%). Most severe acute adverse events were observed after SRT of brain metastases (18 of 265 treatments [6.8%]) (eTable 1 in Supplement 1; Figure 1A). Observed severe acute adverse events include cognitive disturbance, insomnia, intracranial hemorrhage, seizure, fatigue, dyspnea, esophagitis, pain, and spinal fracture (eTable 1 in Supplement 1). Acute grade 4 adverse events were cerebral edema (5 treatments), intracranial hemorrhage (1 treatment), and upper gastrointestinal hemorrhage (1 treatment) (eTable 1 in Supplement 1). Severe acute adverse events were more frequent after intracranial vs extracranial SRT without reaching statistical significance (odds ratio [OR], 1.88; 95% CI, 0.83-4.27) (Table 3). Severe acute adverse events were most frequently observed in patients where SRT was added to BRAF/MEKi (6 of 39 patients [15.4%]), aPD-(L)1 plus anticytotoxic T-lymphocyte-associated protein 4 (4 of 44 patients [9.1%]), and single-agent aPD-(L)1 (15 of 263 patients [5.7%]) (Figure 1A). Uninterrupted BCT during SRT was not significantly associated with increased severe acute adverse events (no BCT during SRT: 7 of 155 patients [4.5%]; continued SRT: 20 of 347 patients [5.8%]; OR, 1.29; 95% CI, 0.54-3.12) (Table 3).

**Table 3.** Univariate Regression Analysis for Factors Associated With Severe Adverse Events

Characteristics	Acute severe adverse events (>grade 3) (n = 27)		Late severe adverse events (>grade 3) (n = 29)	
	No./total No. (%)	OR (95% CI)	No./total No. (%)	OR (95% CI)
Biological cancer therapy during SRT				
Yes	20/347 (5.8)	1.29 (0.54-3.12)	24/311 (7.7)	2.32 (0.87-6.22)
No (treatment interrupted or started after SRT)	7/155 (4.5)	1 [Reference]	5/144 (3.5)	1 [Reference]
ECOG-PS prior to SRT treatment				
0-1	26/456 (5.7)	1 [Reference]	26/415 (6.3)	1 [Reference]
2-4	1/34 (2.9)	0.50 (0.07-3.81)	3/29 (10.3)	1.73 (0.49-6.08)
Charlson Comorbidity Index Score				
0-3	16/260 (6.2)	1 [Reference]	18/234 (7.7)	1 [Reference]
>3	11/246 (4.5)	0.71 (0.32-1.57)	11/225 (4.9)	0.62 (0.28-1.34)
Histologic type				
Melanoma	17/181 (9.4)	1 [Reference]	9/167 (5.4)	1 [Reference]
Other	10/325 (3.1)	0.80 (0.62-1.03)	20/292 (6.8)	0.89 (0.71-1.11)
Location SRT treatment				
Cranial	18/265 (6.8)	1.88 (0.83-4.27)	19/234 (8.1)	1.90 (0.86-4.18)
Extracranial	9/241 (3.7)	1 [Reference]	10/225 (4.4)	1 [Reference]
Gross tumor volume, mL				
Cranial				
≤2	7/128 (5.5)	0.53 (0.20-1.41)	10/114 (8.8)	1.23 (0.47-3.23)
>2	12/123 (9.8)	1 [Reference]	8/110 (7.2)	1 [Reference]
Extracranial				
≤10	2/104 (1.9)	0.38 (0.07-2.03)	4/100 (4.0)	0.73 (0.19-2.82)
>10	5/103 (4.9)	1 [Reference]	5/93 (5.4)	1 [Reference]
Prescribed dose (BED10), Gy				
Cranial			NA	NA
>60	7/159 (4.4)	0.54 (0.20-1.50)	NA	NA
>100	1/18 (5.6)	0.95 (0.12-7.59)	NA	NA
Extracranial				
>60	5/133 (3.8)	0.36 (0.04-2.94)	NA	NA
>100	1/60 (1.7)	0.99 (0.26-3.77)	NA	NA
Prescribed dose (BED3), Gy				
Cranial				
>100	NA	NA	15/197 (7.6)	0.85 (0.23-3.11)
>250	NA	NA	0	NA
Extracranial				
>100	NA	NA	10/168 (6.0)	1.39 (0.08-25.41)
>250	NA	NA	5/69 (7.2)	1.63 (0.45-5.83)

Abbreviations: BED, biological equivalent dose; ECOG-PS, SRT, Eastern Cooperative Oncology Group Performance Status; OR, odds ratio; SRT, stereotactic radiotherapy.

### Late Adverse Events

Severe late (≥grade 3) adverse events were observed in 29 of 459 patients (6.3%). Observed severe late adverse events include central nervous system necrosis, cerebral edema, cognitive disturbance, nausea, vomiting, pain, dyspnea, pneumonitis, colitis, and fatigue (eTable 2 in Supplement 1). Late grade 4 adverse events were cerebral edema (5 patients), central nervous system necrosis (1 patient), seizure (1 patient), and unknown (1 patient) (eTable 2 in Supplement 1). Prevalence rates of severe adverse events remained low at different time points in follow-up (6 months: 9 of 367 patients; 12 months: 20 of 308 patients; 24 months: 6 of 195 patients) (eTable 3 in Supplement 1). Severe late adverse events were more frequently observed after cranial SRT compared with extracranial SRT (eTable 2 in Supplement 1; Figure 1B); however, there was no association of intracranial SRT vs extracranial SRT with severe late adverse events (OR, 1.90; 95% CI, 0.86-4.18) (Table 3). Severe late adverse events were most frequently observed in patients treated with anti–epidermal growth factor receptor and epidermal growth factor receptor inhibitor (aEGFR/EGFRi; 8 of 44 patients [18.2%]), anti–human epidermal growth factor receptor 2 and human epidermal growth factor receptor 2 inhibitor (3 of 21 patients [14.3%]) and BRAF/MEKi (3 of 35 patients [8.6%]) (Figure 1B). SRT with uninterrupted BCT was not significantly associated with increased severe late adverse events (OR, 2.32; 95% CI, 0.87-6.22) (Table 3).

### Survival Outcomes

**Figure 2.**
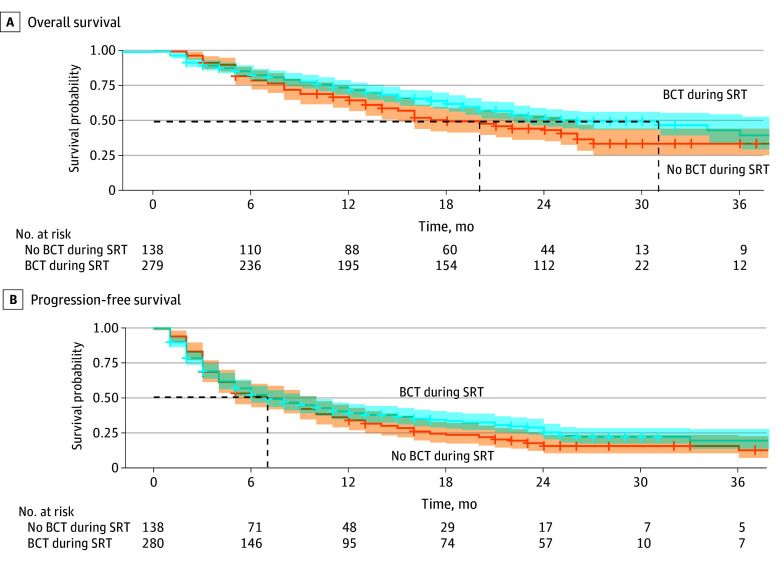
Survival Curves per Biological Cancer Therapy (BCT) Continuation for Overall Survival and Progression-Free Survival The black dashed lines indicate the median overall survival at 20 and 31 months (A) and the median progression-free survival at 7 months (B). SRT indicates stereotactic radiotherapy.

Median OS was 24 months (95% CI, 21-27 months) and cause of death was cancer-related in 28 of 34 cases (82.4%). When BCT was continued during SRT, patients showed improved OS compared with patients whose BCT was paused during or started after SRT (median OS, 31 months; 95% CI, 22-not reached vs 20 months; 95% CI, 15-26 months; *P* = .046) (Figure 2). After adjusting for ECOG PS, Charlson comorbidity index and histologic type of the primary tumor, the difference in OS lost statistical significance (hazard ratio, 0.81; 95% CI, 0.61-1.09; *P* = .17).

Of the 433 patients, 320 (73.9%) had disease progression within 24 months, which was symptomatic in 112 of 320 cases (35.0%). Median PFS was 7 months (95% CI, 6-9 months). There was no difference in PFS between continued BCT vs interruption or delayed start of BCT (median PFS, 7 months [95% CI, 6-10 months] vs 7 months [95% CI, 5-10 months]; *P* = .18) (Figure 2).

## Discussion

Overall, this cohort study found that metastases-directed SRT concurrent with BCT was associated with low rates of severe acute and late adverse events (<10%), indicating that the favorable safety profile of SRT persists in a combined modality treatment setting.^28^ There were no significant differences between patients treated for cranial and extracranial metastases; however, all grade 5 adverse events were observed after cranial SRT. This study provides exploratory information about safety profiles of specific SRT areas in combination with specific BCT drugs, albeit with limited numbers of patients for individual treatment combinations.

SRT using highly focused and escalated radiation doses in a single or few fractions has been associated with unique radiobiological consequences such as increased microvascular damage or change of antitumor immunity compared with conventionally fractionated radiotherapy.^29^ These distinctions have raised concerns about potentially increased risk of adverse events when SRT is combined with BCT, also based on limited preclinical and clinical data.^16,21,28,30^ Our observations confirm an overall low incidence of severe SRT-associated adverse events, underscoring the potential of SRT as locally ablative metastasis-directed treatment in patients with metastatic or oligometastatic cancer treated with concomitant BCT.^16,23,28,31,32^ This favorable safety profile was observed with SRT doses being most frequently less than 100 Gy BED, which is in agreement with the practice of prospective trials and recommendations of clinical practice guidelines in the field of oligoprogressive cancer.^10,33,34^

Concurrent SRT and aEGFR/EGFRi was associated with the highest rate of severe late adverse events (18.2%), especially in the setting of cranial, thoracic, or abdominal SRT. No severe acute adverse events were reported in these patients, indicating the need for long-term follow-up. Previous studies indicated that EGFRi combined with SRT is associated with a low risk (<10%) of severe pulmonary, intraabdominal, or bone adverse events.^16,21^ These studies were characterized by shorter follow-up times and a lack of distinction between acute and late adverse events. Moreover, (late) safety data on the combination of aEGFR/EGFRi and SRT is only documented in a limited number of patients, indicating the need for prospective and detailed toxicity assessment.^23^

No severe adverse events were observed for the combination of antivascular endothelial growth factor (aVEGF) and SRT, suggesting that the combination of SRT with aVEGF may not be an absolute contraindication. aVEGF treatment was not paused during SRT and no SRT dose reduction was reported. Previous data have suggested that the combination of aVEGF and SRT might be associated with increased risk of severe adverse events, especially within the abdominal and thoracic region.^16,35,36^ In our study, a limited number of patients (16 patients) had been treated with this combination for liver metastases, indicating that abdominal SRT with concurrent aVEGF is only carefully performed in routine practice.

Overall, both acute and late severe adverse events were more frequently observed after SRT for intracranial metastases; however, differences were small and not statistically significant. Rates of acute and late severe adverse events were low and very similar across all extracranial metastases locations, indicating a favorable safety profile of SRT irrespective of metastasis location, also in the setting of concurrent BCT.

To potentially minimize adverse events, it remains unknown whether it is beneficial to interrupt or to delay the start of BCT during SRT, especially because of the long half-life time of many mAbs and ICIs (range, 24 hours to 50 days) and prolonged immune system activation.^23^ This results in heterogeneity in daily clinical practice, with potential risks of increased adverse events if BCT is continued or disease flare if BCT is interrupted during SRT.^37^ In our study, a numerical increased risk for severe acute or late adverse events was observed for patients who continued BCT during SRT, which was not statistically significant. Regarding efficacy, patients who continued BCT had longer OS. However, this difference might be explained by variations in disease extent, patients’ performance status, and comorbidities, which may influence the physician’s decision to either continue or interrupt BCT. A previous retrospective study also did not observe significant differences in severe adverse events, PFS, and OS when BCT was interrupted or continued during SRT.^31^ Another retrospective study on patients with lung cancer with acquired resistance to EGFR-tyrosine kinase inhibitor therapy reported a 23% disease flare rate after a median of 8 days after stopping tyrosine kinase inhibitors (61 patients).^38^ This discrepancy might be explained by the limited number of patients in this subgroup in the present cohort study or the addition of SRT to the patients in the present cohort study. Additionally, in the present study, we collected data on PFS rather than specifically on disease flare. Moreover, most patients received ICIs and mAbs with long half-lives, where treatment interruption for less than 1 month might make no difference. Our findings suggest that pausing BCT during SRT is not associated with accelerated disease progression. However, due to limited data, it remains important to be cautious of disease flare.

### Strengths and Limitations

The collection of data in a large international patient cohort with detailed capturing of adverse events over 24 months of follow-up are strengths of this study. Limitations are heterogeneity of the data, which only allowed exploratory analyses in subcohorts. Because adverse events were only recorded for the combination of BCT and SRT and not also for single-modality treatments, it is difficult to assess the magnitude of possible added adverse events due to treatment interactions. For intracranial metastases, cerebral edema, seizure, and cognitive disturbance could also occur due to radiation necrosis or progression. Intracranial hemorrhage may result from the combination of stereotactic radiotherapy and BRAF/MEKi, but could also reflect the inherent hemorrhagic tendency of melanoma.

Another limitation of the study was the risk of biases. First, previous therapies could have caused some of the adverse events reported. Partly, our study compensates for this by only reporting adverse events within the current SRT field. Second, adverse events were graded by the treating physician, and not in the form of patient-reported outcome measures, which could potentially have led to an underreporting of adverse events. Third, melanoma was the most common primary tumor in our cohort (37.0%), which is not representative of the incidence of cancer types in the general population; this might be explained by BCT as standard of care for the treatment of melanoma. Furthermore, the indication for SRT treatment is unknown, which could introduce bias in the observed adverse events.

## Conclusions

In conclusion, this multicenter prospective registry study observed a low risk (<10%) of severe acute or late adverse events following concurrent treatment with metastases-directed SRT and BCT. Uninterrupted treatment with BCT during SRT was not associated with significantly increased risk of severe adverse events. These findings may guide the design of patient-individual combined-modality treatment strategies.

## Notes

Supplement 1. eTable 1. Observed acute severe (≥G3) toxicity per treatment site and biological cancer therapy (BCT) type eTable 2. Observed late severe (≥G3) toxicity per treatment site and biological cancer therapy (BCT) type eTable 3. Prevalence of severe toxicity at different follow-up time points

Supplement 2. Data Sharing Statement
