# Errors in Figure and Byline

**DOI:** 10.1001/jamanetworkopen.2026.2380

**Published:** 2026-02-23

In the Original Investigation titled “Adverse Events After Metastases-Directed Stereotactic Radiotherapy and Biological Cancer Therapy,”^1^ published January 14, 2026, there were errors in Figure 1 and the byline. In Figure 1, the number of events for anti-programmed death-ligand 1 antibody therapy for the brain was incorrectly reported as 10 of 11 events instead of 10 of 111 events. Author Stephanie G.C. Thoma’s name in the byline was updated from Stephanie G.C. Kroeze; this change was also made to the author contributions section. This article has been corrected.^1^

## References

[1] Looman EL, Thoma SGC, Schaule J, . Safety of concurrent metastases-directed stereotactic radiotherapy and biological cancer therapy. JAMA Netw Open. 2026;9(1):e2553809. doi:10.1001/jamanetworkopen.2025.53809 41533377 PMC12805445

