# Supplementary material for: Adverse Events After Metastases-Directed Stereotactic Radiotherapy and Biological Cancer Therapy
Source: JAMA Netw Open. 2026 Jan 14;9(1):e2553809. doi: 10.1001/jamanetworkopen.2025.53809 (PMC12805445; doi:10.1001/jamanetworkopen.2025.53809)
Supplement: Supplement 1. — eTable 1. Observed acute severe (≥G3) toxicity per treatment site and biological cancer therapy (BCT) type eTable 2. Observed late severe (≥G3) toxicity per treatment site and biological cancer therapy (BCT) type eTable 3. Prevalence of severe toxicity at different follow-up time points [file jamanetwopen-e2553809-s001.pdf]

Supplemental Online Content

Looman EL, Thoma SGC, Schaule J, et al. Safety of concurrent metastases-directed stereotactic radiotherapy and biological cancer therapy. *JAMA Netw. Open*. 2026;9(1):e2553809.
doi:10.1001/jamanetworkopen.2025.53809

eTable 1. Observed acute severe toxicity per treatment site and biological cancer therapy (BCT) type

eTable 2. Observed late severe toxicity per treatment site and biological cancer therapy (BCT) type

eTable 3. Prevalence of severe toxicity

This supplemental material has been provided by the authors to give readers additional information about their work.

© 2025 Looman EL et al. *JAMA Netw. Open*.

eTable 1. Observed acute severe toxicity per treatment site and biological cancer therapy (BCT) type.

Acute severe toxicity (>G3) (N=27)										
	**Overall**	**aPD-(L)1**	**aPD-(L)1 + aCTLA4**	**BRAF/MEKi**	**aEGFR/EGFRi**	**mTKI**	**ALKi**	**aVEGF**	**aHER2/HER2i**	**Other**
**Brain (N (%))**										
Overall	18/265	10/111	2/36	5/33	0/20	0/19	1/15	0/7	0/14	0/10
Other; grade 5	1	1								
Intracranial hemorrhage; grade 5	2	1		1						
Cerebral edema; grade 4	5	4					1			
Intracranial hemorrhage; grade 4	1			1						
Cognitive disturbance; grade 3	1	1								
Insomnia; grade 3	1	1								
Intracranial hemorrhage; grade 3	3	2	1							
Intracranial hemorrhage + seizure; grade 3	1			1						
Fatigue + other; grade 3	2		1	1						
Seizure; grade 3	1			1						
**Thorax (N (%))**										
Overall	3/123	3/72	0/3	0/2	0/21	0/9	0/2	0/4	0/3	0/7
Dyspnea; grade 3	1	1								
Esophagitis; grade 3	1	1								
Other; grade 3	1	1								
**Abdomen (N (%))**										
Overall	3/53	1/31	2/3	0/2	0/6	0/1	0/0	0/5	0/2	0/3
Upper gastrointestinal hemorrhage; grade 4	1	1								
Abdominal pain; grade 3	1		1							
Other; grade 3	1		1							
**Pelvis (N (%))**										
Overall	2/41	1/31	0/1	0/1	0/2	1/2	0/0	0/0	0/3	0/1
Spinal fracture; grade 3	1					1				
Other; grade 3	1	1								
**Other (N (%))**										
Overall	1/24	0/18	0/1	1/1	0/0	0/1	0/1	0/0	0/0	0/2
Bone pain; grade 3	1			1						

© 2025 Looman EL et al. *JAMA Netw. Open*.

eTable 2. Observed late severe toxicity per treatment site and biological cancer therapy (BCT) type.

Late severe toxicity (>G3) (N=29)										
	**Overall**	**aPD-(L)1**	**aPD-(L)1 + aCTLA4**	**BRAF/MEKi**	**aEGFR/EGFRi**	**mTKI**	**ALKi**	**aVEGF**	**aHER2/HER2i**	**Other**
**Brain (N (%))**										
Overall	19/234	5/96	2/31	3/30	4/17	0/19	1/13	0/6	3/13	1/9
Central nervous system necrosis + intracranial hemorrhage; grade 5	1			1						
Intracranial hemorrhage; grade 5	1			1						
Central nervous system necrosis + seizure; grade 4	1				1					
Cerebral edema; grade 4	5	2	1				1		1	
Unknown; grade 4	1				1					
Central nervous system necrosis + cerebral edema + cognitive disturbance; grade 3	2				1				1	
Central nervous system necrosis + cerebral edema + nausea + vomiting; grade 3	1		1							
Central nervous system necrosis + other; grade 3	1			1						
Cerebral edema; grade 3	2	1								1
Cognitive disturbance; grade 3	1	1								
Unknown	3	1			1				1	
**Thorax (N (%))**										
Overall	6/115	2/65	0/3	0/2	3/20	1/9	0/2	0/4	0/3	0/7
Bone pain; grade 3	1					1				
Dyspnea; grade 3	2	1			1					
Pneumonitis + dyspnea + non-cardiac chest pain; grade 3	1	1								
Other; grade 3	2				2					
**Abdomen (N (%))**										
Overall	3/49	2/30	0/2	0/2	1/5	0/1	0/0	0/5	0/2	0/2
Colitis; grade 3	1	1								
Fatigue; grade 3	1				1					
Unknown	1	1								
**Pelvis (N (%))**										
Overall	1/37	1/27	0/1	0/1	0/2	0/2	0/0	0/0	0/3	0/1
Other; grade 3	1	1								
**Other (N (%))**										
Overall	0/24	0/19	0/1	0/0	0/0	0/1	0/1	0/0	0/0	0/2

© 2025 Looman EL et al. *JAMA Netw. Open*.

eTable 3. Prevalence of severe toxicity

	Severe toxicity events (N)	Patients at risk (N)*	Prevalence
**Follow-Up 3 Months**	27	425	6.4%
**Follow-Up 6 Months**	9	367	2.5%
**Follow-Up 12 Months**	20	308	6.5%
**Follow-Up 24 Months**	6	195	3.1%
* alive and follow-up available			

© 2025 Looman EL et al. *JAMA Netw. Open*.
