# Supplementary material for: Adverse Events After Metastases-Directed Stereotactic Radiotherapy and Biological Cancer Therapy
Source: JAMA Netw Open. 2026 Jan 14;9(1):e2553809. doi: 10.1001/jamanetworkopen.2025.53809 (PMC12805445; doi:10.1001/jamanetworkopen.2025.53809)
Supplement: Supplement 2. — Data Sharing Statement [file jamanetwopen-e2553809-s002.pdf]

Data Sharing Statement

Looman. Safety of Concurrent Metastases-Directed Stereotactic Radiotherapy and Biological Cancer Therapy. *JAMA Netw Open*. Published January 14, 2026.
doi:10.1001/jamanetworkopen.2025.53809

Data
Data available: No
